# Pulmonary Alveolar Microlithiasis in a Middle-Aged Man Presenting With Respiratory Failure: A Case Report and Review of the Literature

**DOI:** 10.7759/cureus.54942

**Published:** 2024-02-26

**Authors:** Allen C Omo-Ogboi, Joyce Ederhion, Asad Ur Rehman, Olanrewaju Ogunleye, Jaiyeola Thomas-Ogunniyi

**Affiliations:** 1 Department of Pathology and Laboratory Medicine, The University of Texas Health Science Center, Houston, USA; 2 Department of Neuroscience and Immunology, University of Roehampton, London, GBR; 3 Internal Medicine, University of Benin, Benin City, NGA; 4 Department of Radiology, The University of Texas Health Science Center, Houston, USA

**Keywords:** type ii alveolar pneumocytes, intra-alveolar calcium phosphate microliths, bilateral orthotropic lung transplant, slc34a2, pulmonary alveolar microlithiasis

## Abstract

Pulmonary alveolar microlithiasis (PAM) is an autosomal recessive disease of the lung, characterized by diffuse deposits of intra-alveolar calcium phosphate microliths. It usually affects both sexes, presenting mostly in the second and third decades. The clinical course is highly variable, ranging from being asymptomatic to respiratory failure. PAM is usually diagnosed after careful clinical, radiological, and pathological evaluation, usually when patients present for other medical purposes. Here, a case of PAM in a middle-aged man presenting with acute-on-chronic hypoxemic respiratory failure is reported, with a review of the literature.

## Introduction

Pulmonary alveolar microlithiasis (PAM) is an autosomal recessive disease of the lung, characterized by diffuse deposits of intra-alveolar calcium phosphate microliths [1]. It affects both sexes and has been reported in all ages, occurring mostly in the second and third decades.

PAM is caused by a genetic mutation in solute carrier family 34 members 2 (*SLC34A2*), and it is located on chromosome 4p15.2, which is responsible for encoding sodium-phosphate co-transporter type IIb located in type II alveolar pneumocytes [2]. *SLC34A2* plays an important role in phosphate ions transportation from pulmonary alveoli into alveolar type II pneumocytes [2]. The mutation of *SLC34A2* causes alveolar type II dysfunction which eventually leads to cellular phosphate accumulation and microlith deposition in pulmonary alveolar spaces [2]. The clinical course is highly variable, ranging from being asymptomatic to respiratory failure [1]. PAM is usually diagnosed after careful clinical, radiological, and pathological evaluation, usually when patients present for other medical purposes. This report describes the case of PAM in a middle-aged man presenting with acute-on-chronic hypoxemic respiratory failure.

## Case presentation

A 38-year-old man with a past medical history of interstitial lung disease of unknown etiology, chronic hypoxic respiratory failure on home oxygen, and pulmonary hypertension was admitted for acute-on-chronic hypoxemic respiratory failure. The family reported a week-long history of shortness of breath on exertion and increased oxygen requirement to 6-8L above his baseline of 3L only at night and worsening in his daily functions over the past six months. 

**Figure 1:**
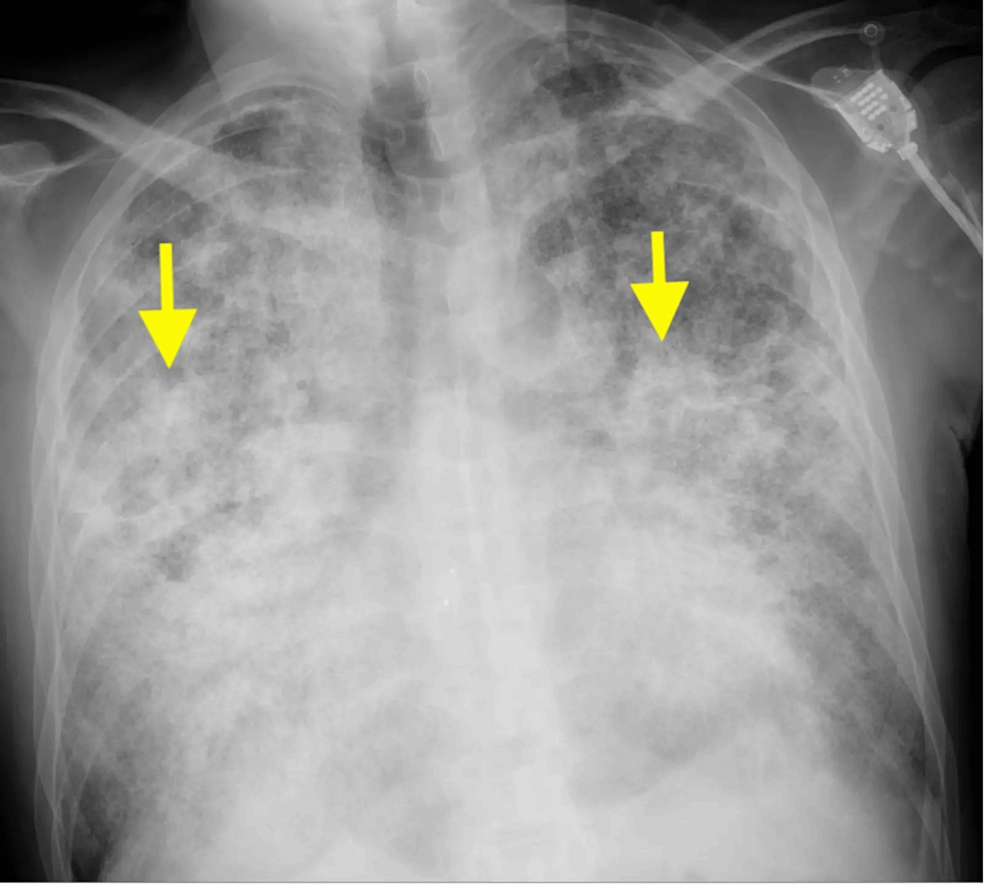
Chest radiograph showing fine sand-like infiltrates (yellow arrows) in both lungs

**Figure 2:**
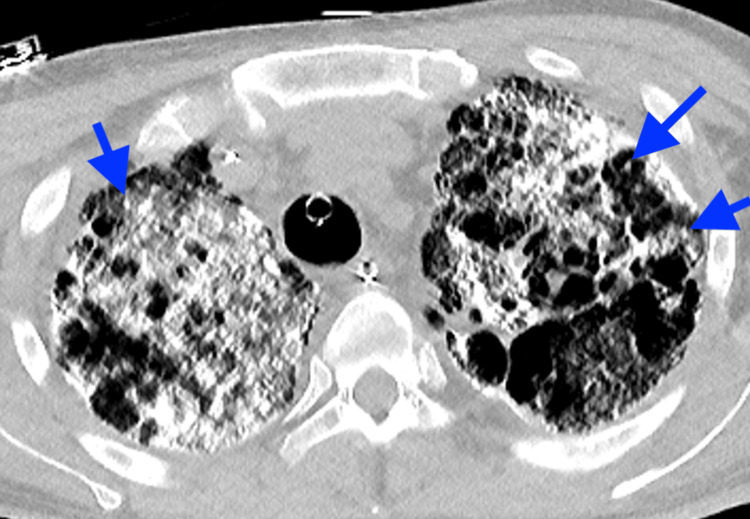
High-resolution axial chest CT showing diffuse, coalescing calcified micronodules (blue arrows) in both lungs, with subpleural and peri-bronchial distribution, interspersed with ground glass opacities

**Figure 3:**
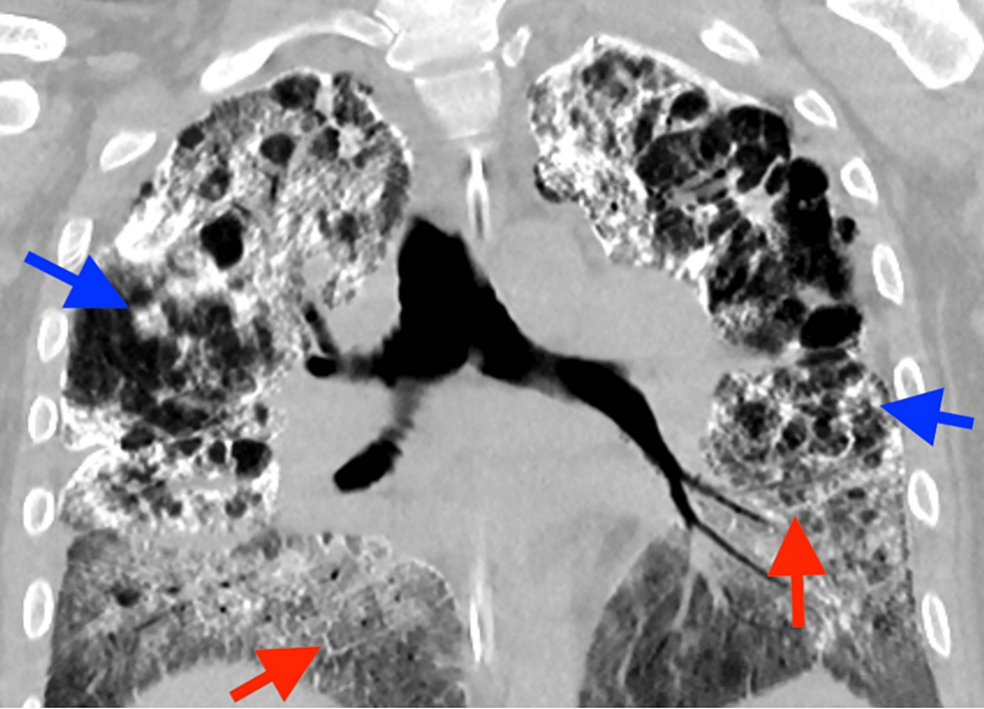
High-resolution coronal chest CT showing diffuse, coalescing calcified micronodules (blue arrows) and interlobular septal thickening (red arrows)

Chest radiograph showed diffuse bilateral calcified micronodules with relative sparing of the apices and costophrenic sulci (Figure 1). High-resolution computed tomography (HRCT) performed showed diffuse, coalescing, sand-like, calcified micronodules of both lungs with subpleural and peri-bronchial distribution, interspersed with ground glass opacities. In addition, there were pleural and interlobular septal calcifications, tiny subpleural cysts, and scattered apical bullae (Figures 2, 3).

The family also reported that 14 years prior, during the flu pandemic, he developed a viral illness and ended up on the ventilator with a long ICU stay. There was no known family history of chronic interstitial lung disease. The decision was taken at the multidisciplinary interstitial lung disease conference that he had PAM and would benefit from a bilateral orthotropic lung transplant, which was subsequently performed.

**Figure 4:**
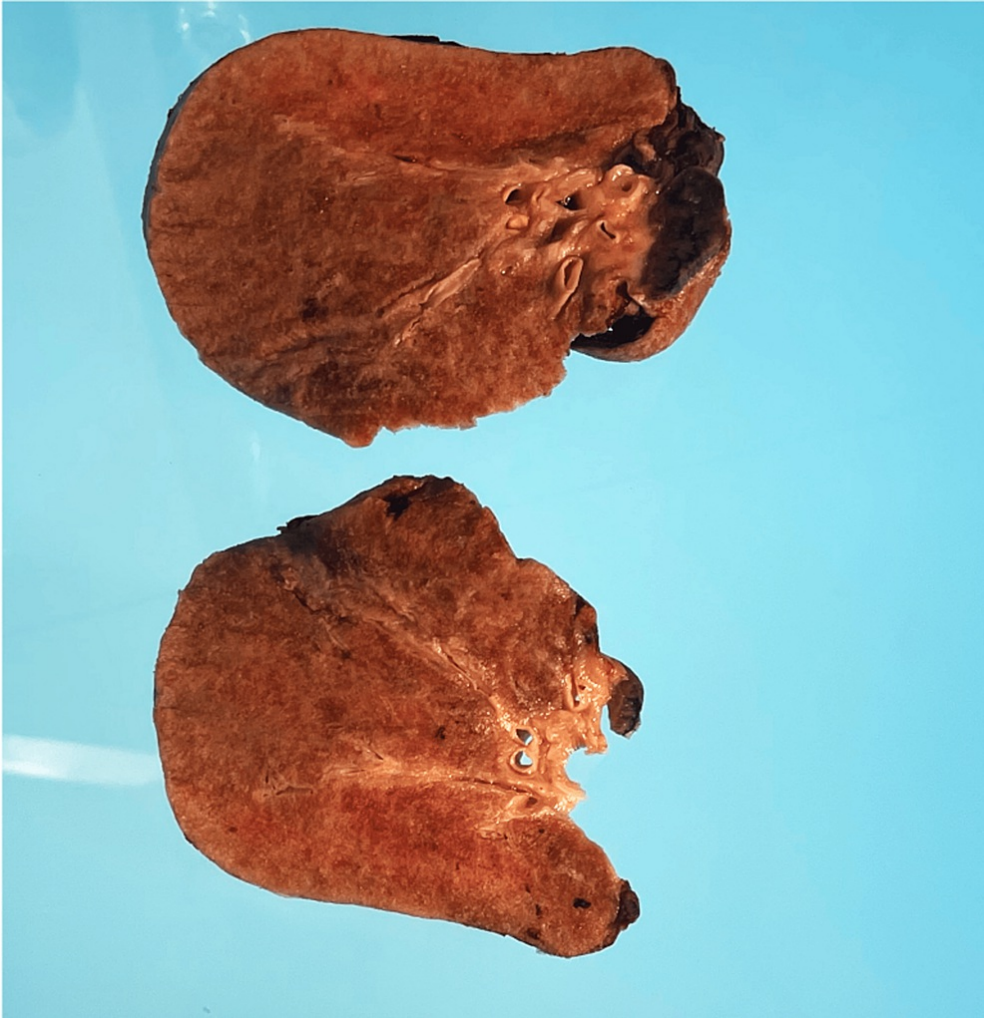
Cross sections of both lungs showing solid, firm parenchyma.

Gross examination showed bilateral heavy lungs, weighing (right lung) 1732 gm and (left lung) 1442 gm. The pleural surfaces of both lungs were granular and pale. The cut sections were solid and very firm, tan-brown, and gritty to cut, demonstrating a granular sandy feel to touch with diffuse areas of fibrotic parenchyma and fine sand-like material (Figure 4). Focal pockets of aerated spaces were identified in the upper lobes. 

**Figure 5:**
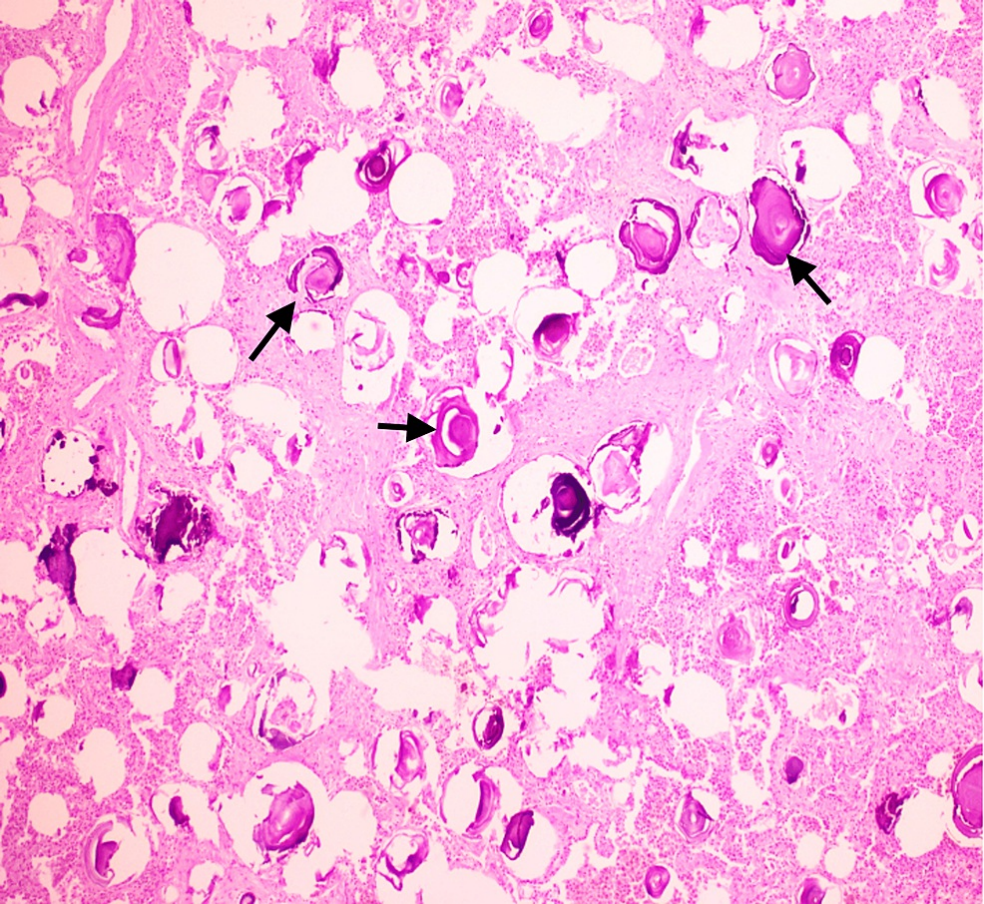
Numerous heavily calcified lamellar bodies (black arrows) seen within the alveolar spaces (H&E x 200)

**Figure 6:**
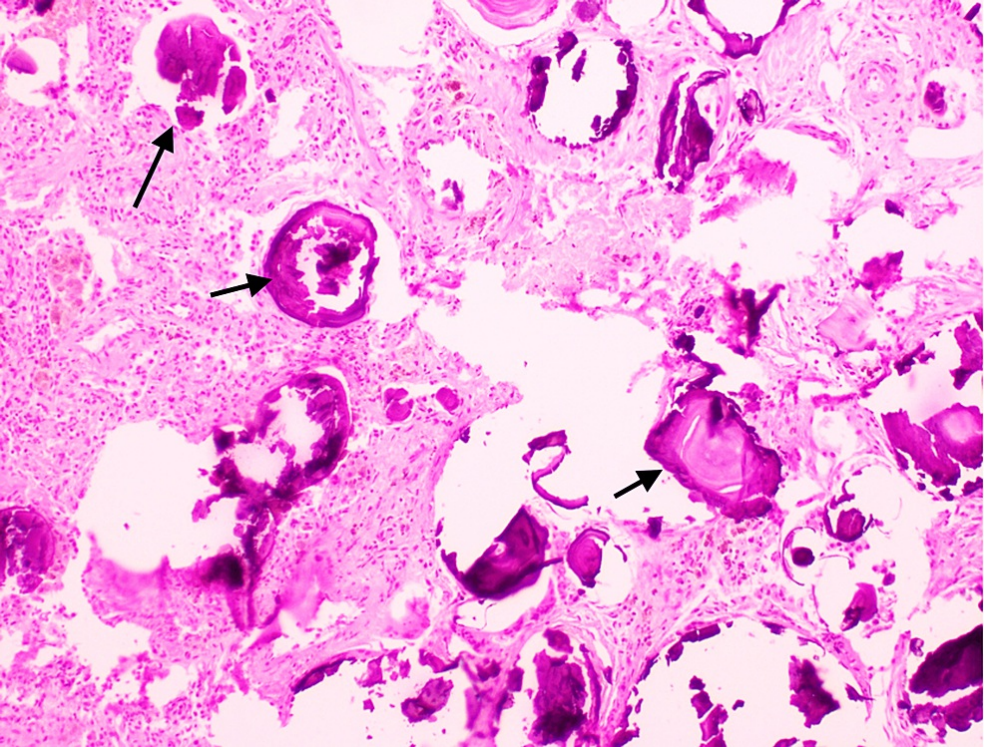
Numerous heavily calcified lamellar bodies (black arrows) seen within the alveolar spaces (H&E x 400)

Microscopic examination of both lungs showed numerous heavily calcified lamellar bodies within alveolar spaces (Figures 5, 6). Given the clinical, radiologic, and pathologic findings, these features were consistent with PAM.

## Discussion

PAM of the lungs is a rare entity, first described in 1868 by Marcello Malpighi [1,3]. Since then, over 1000 cases have been reported in the literature [1,4]. Although PAM is rare, cases have been reported in all continents with the highest prevalence of cases seen in Asia Minor (576 cases; 56.3%) and Europe (285 cases; 27.8%) [4]. PAM has both genetic and sporadic cases, with familial history reported in a frequency ranging from 32% to 61% [4]. Our case has no family history of PAM or chronic interstitial lung disease. The genetic cases are associated with a rare autosomal recessive disease with high penetrance, and a history of consanguinity is common in these cases [1,5].

There is no gender predilection in genetic cases. Although it has been reported that sporadic cases have a slight male predominance [1,4], a slight female predominance has also been reported by some studies [2]. PAM has been reported in all ages. While it presents mostly in the second and third decades [1], it is also seen in the pediatric age group [6] and in infants [1]. Some studies have reported that PAM usually occurs among families in horizontal patterns, affecting siblings more frequently than vertical patterns affecting parents and children [2,7]. Some cases are asymptomatic prior to presentation for other medical conditions [2], but others present with respiratory failure, as seen in our case. 

On gross examination, the affected lungs are usually very large, heavy, and dusky [2]. Serial sectioning of the affected lung tissue reveals diffuse, fine, granular cut surface and calcification with gritty sensation [2,4]. Microscopic examination is the gold standard for diagnosis, which is usually done after careful clinical and radiologic evaluation [2,8]. Microscopic examination demonstrates classical intra-alveolar lamellated calcified microliths, composed of mostly calcium admixed with other minerals [2,8]. PAM may have extra-pulmonary calcium deposition, particularly in the testes, which may result in testicular atrophy, azoospermia, tumorigenesis, or other testicular pathologies [2]. Other extra-pulmonary involvement such as kidney, lumbar sympathetic chain, and cardiac have also been reported [2].

Genetic testing can be done as a screening test for family members of affected patients to detect the *SLC34A2 *gene mutation [2]. Treatment in most cases remains supportive, including supplemental oxygen therapy, and for patients presenting with respiratory failure, bilateral lung transplantation is usually the last resort as seen in our case [2,9].

## Conclusions

A case of PAM in a middle-aged man presenting with respiratory failure was described here. The diagnosis of PAM was made after careful clinical, radiological, and pathological evaluation. Management of PAM depends on the severity of the presentation since presentation varies from asymptomatic to respiratory failure. Sex predominance is still not entirely clear, and more studies have to be done in the future.
